# PCBS: an R package for fast and accurate analysis of bisulfite sequencing data

**DOI:** 10.1093/bioinformatics/btae593

**Published:** 2024-10-04

**Authors:** Kathryn Lande, April E Williams

**Affiliations:** The Razavi Newman Integrative Genomics and Bioinformatics Core Facility, The Salk Institute for Biological Studies, La Jolla, CA 92037, United States; The Razavi Newman Integrative Genomics and Bioinformatics Core Facility, The Salk Institute for Biological Studies, La Jolla, CA 92037, United States

## Abstract

**Motivation:**

Whole-genome bisulfite sequencing is a powerful tool for analyzing chromatin methylation genome-wide, but analysis of whole-genome bisulfite data is slow, inflexible, and often inaccurate.

**Results:**

We developed PCBS (Principal Component BiSulfite), a computationally efficient R package for Whole Genome Bisulfite Sequencing analysis that demonstrates remarkable accuracy and flexibility compared to current tools. PCBS identifies differentially methylated loci, differentially methylated regions, and offers novel functionality that allows for more targeted methylation analyses. PCBS uses minimal computational resources; a complete pipeline in mouse can run on a local RStudio instance in a matter of minutes.

**Availability and implementation:**

PCBS is an R package available under a GNU GPLv3 license on GitHub: https://github.com/katlande/PCBS and CRAN: https://CRAN.R-project.org/package=PCBS. Instructions for use are available at: https://katlande.github.io/PCBS/.

## 1 Introduction

DNA methylation plays important roles in a variety of basic biological functions such as gene splicing, transcription, and chromosomal stability, as well as in numerous disease states including cancer, autoimmune, and neurodevelopmental diseases (Robertson 2005, Jones 2012, Grolaux *et al.* 2022). Whole Genome Bisulfite Sequencing (WGBS) is one of the most powerful tools for assessing methylation states genome-wide.

The current analysis paradigm for WGBS generally involves identifying a small number of significantly differentially methylated loci (DMLs) and/or differentially methylated regions (DMRs) in order to classify the differences in methylation between experimental conditions. While these sites and regions are useful, focusing on them exclusively risks discarding a large amount of biologically relevant information and diminishes the flexibility and power granted by a whole genome dataset. However, because analyses of WGBS data are plagued by slow computational times that root from the massive sizes of whole genome data, many researchers ultimately opt to focus just on these selected sites. Here, we introduce Principal Component BiSulfite (PCBS): a novel, user-friendly, and computationally efficient R package for analyzing WGBS data holistically.

PCBS is built on the simple premise that if a principal component analysis (PCA) strongly delineates samples between two conditions, then the value of a methylated locus in the eigenvector of the delineating principal component (PC) will be larger if that locus is highly different between conditions. Thus, eigenvector values, which can be calculated quickly for even a very large number of sites, can be used as a score that roughly defines how much any given locus contributes to the variation between two conditions. This premise has been previously described for analysis of methylation array data (Zheng *et al.* 2023), but to our knowledge it has never been packaged for the analysis of WGBS data. Herein, we provide several new tools for analyzing WGBS data under this paradigm and provide a proof of concept that PCBS matches or outperforms other commonly used tools in speed and accuracy metrics when used on real and simulated data. While the present paper focuses mostly on benchmarking PCBS’s DML and especially DMR calling functionality against other softwares, details of its more holistic functionality can be found in our vignettes (see: Availability and implementation).

## 2 Materials and methods

PCBS requires the sequencing depth and percent methylation for each locus in each sample, provided in a single data frame with two columns per sample. We offer a script that can convert the output of a Bismark (Krueger and Andrews 2011) alignment pipeline into PCBS input file format and also provide an example input file called eigen in the data of the PCBS R package.

### 2.1 Test data

To test the speed and accuracy of PCBS, we used archived wildtype mouse WGBS samples from (Cole *et al.* 2017), representing methylation in four young and four old individuals. Separately, we considered three smaller, entirely simulated test genomes with differing amounts of true variable sites to assess accuracy (Supplementary Fig. S1A and B). Each simulated genome is 24 megabases in total length, with 1.2 million measured methylated loci across three chromosomes. The simulated genomes have roughly 30x coverage. For the purposes of accuracy testing, we use the simulated genomes as these have known true variable sites. For all other purposes including measurements of computing time, we use the archived mouse data aligned to mm10.

### 2.2 Simulated genomes

Three simulated genomes of equal length were generated with different numbers of true variable sites to reflect low, medium, and high variation comparisons. 8358, 18 774, and 37 192 “true” DMLs were added to each genome, respectively. These DMLs were primarily assigned to “true” DMR regions of randomly assigned lengths between 100 and 4000 bp (45, 106, and 192 true DMRs in the low, medium, and high variation respectively). 1000, 3000, and 6000 stray differential loci were additionally added to each set. These stray DMLs reflect real WGBS data, which we have observed to contain a substantial amount of differentially methylated loci outside of DMRs. In this case, we arbitrarily opted for about 15% of our simulated true sites to be stray DMLs. Each differential locus was randomly assigned as either hypo- or hyper-methylated. They were also randomly assigned a 25%, 50%, or 75% modifier, representing low-, medium-, and high-intensity methylation differences between treatment and control samples.

Per simulation, three treatment and three control samples were generated for each of the three genome variation levels described above, and sites were seeded with a random percent methylation across all samples. The values of each locus’ percent methylation were pulled from a normal distribution [μ = 0.5, SD = 0.1, max = 0.75, min = 0.25] for NS sites, [μ = 0.55, SD = 0.1, max = 0.85, min = 0.35] for hyper sites, and [μ = 0.45, SD = 0.1, max = 0.65, min = 0.15] for hypo sites. The randomly assigned intensity modifiers were applied to each “true” site, so that there was a relatively equal mix of high, medium, and low intensity percent methylation difference represented in each simulated genome.

## 3 Results

### 3.1 Differentially methylated loci

**Figure 1.**
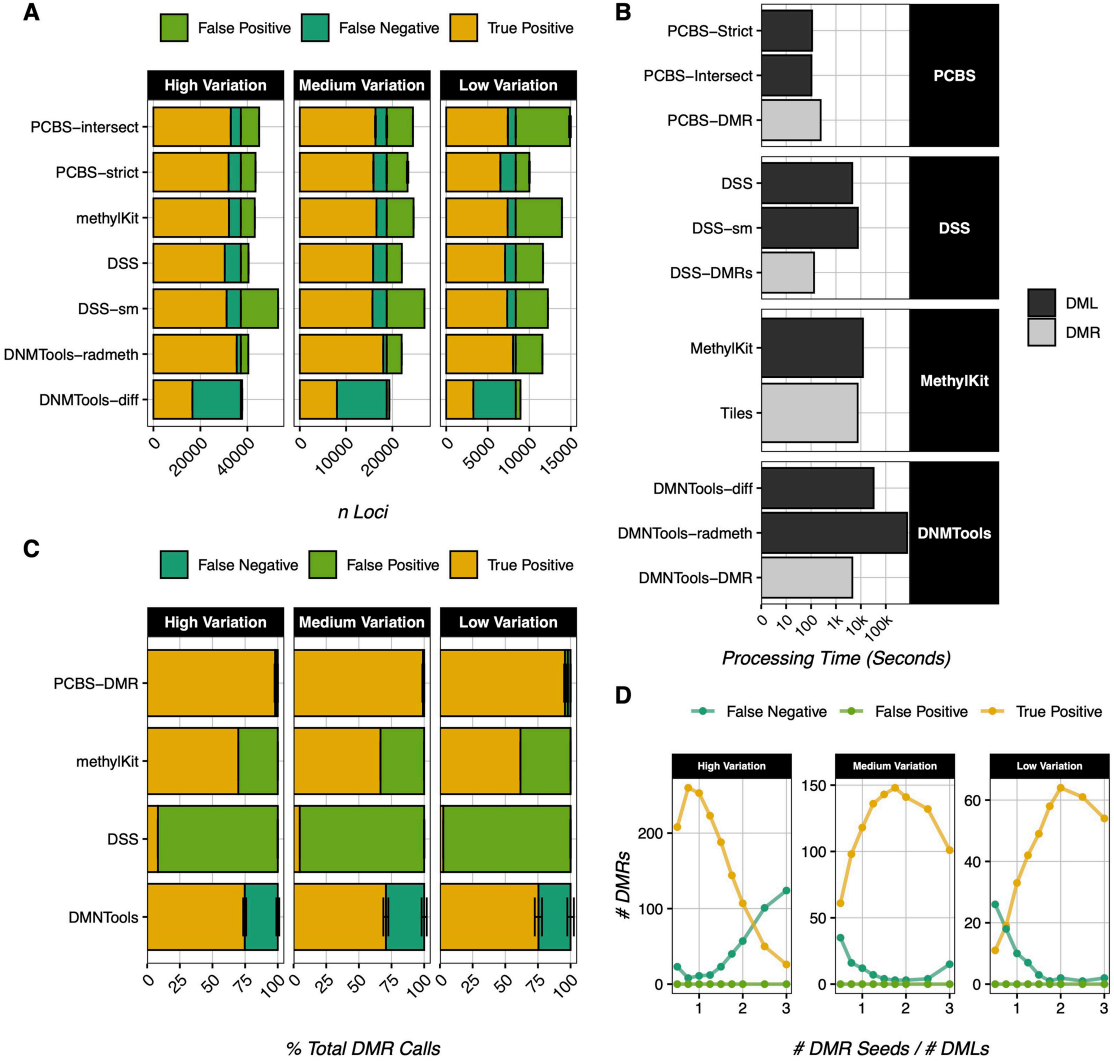
PCBS accuracy and speed testing. (A) DML calling error rates of common WGBS software and PCBS in 127 iterations of 3 treatment versus 3 control samples for three simulated genomes of high, medium, and low variation; standard error of each call type is denoted with error bars. All DML calls use default software parameters. (B) Processing times of DML and DMR calling in common WGBS software and PCBS, on a single CPU with 8 GB RAM. All calls use default software parameters, and are run on eight archived mouse samples from Cole *et al.* (2017) aligned to mm10 at ∼30X coverage. (C) DMR calling error rates of common WGBS software and PCBS in 127 iterations of 3 treatment versus 3 control samples for three simulated genomes of high, medium, and low variation; standard error of each call type is denoted with error bars. DMR calls are determined to be true positives if they overlap any true DMR region. All DMR calls use default software parameters. (D) PCBS DMR calling accuracy under default parameters as a function of input seed number in one iteration of three treatment versus three control samples for three simulated genomes of high, medium, and low variation.

Most WGBS analysis software focuses on calling differentially methylated loci (DMLs) by applying statistical tests such as beta-binomial distributions (Dolzhenko and Smith 2014, Feng and Wu 2019) or logistic regressions (Akalin *et al.* 2012) across samples at all sequenced sites. PCBS does not do this, and opts simply to rank loci by their eigenvector score. While this does not return locus-level *P*-values, a simple rank cut-off performs comparably to software using the aforementioned methodology when identifying DMLs (Fig. 1A), as rank order is strongly correlated to “true” DML sites in simulated datasets (Supplementary Fig. S2A). The optimal cut-off for DML calling occurs just above the inflection point on a plot of locus rank versus absolute locus eigenvector score (Supplementary Fig. 2B). PCBS offers two modes for estimating this cut-off. In most cases, we recommend the “intersect” method, where the rank cut-off is defined as the intersection between the linear line of best fit for the highest-scoring sites (true variation), and that of the lowest-scoring sites (background noise). For very low variation datasets, we also offer the “strict,” method, which functions similarly, but takes the halfway point between PCBS-intersect and the maximum rank value of the true variation line of best fit as the cut-off instead. Moreover, while DML calling accuracy is similar across software including PCBS, PCBS requires the fewest computational resources by a large margin (Fig. 1B).

### 3.2 Differentially methylated regions

DMRs are a cornerstone of WGBS analysis, and are generally defined as regions containing a number of DMLs (Campagna *et al.* 2021, Peters *et al.* 2021). Common DMR callers may look for enrichment in genomic bins (Akalin *et al.* 2012), by merging nearby significant loci and identifying regions above a certain threshold of percent significant sites (Feng and Wu 2019), or by using hidden Markov models (Song *et al.* 2013). However, PCBS uses an entirely novel algorithm to identify DMRs. It works broadly by taking a user-defined rank cut-off, wherein loci above this rank are extracted as “seeds.” It then compares the median eigenvector scores of regions around these seeds against random local background regions in permutations (Supplementary Fig. S3A). Because we expect many of the loci in a single “true” DMR region to be selected as seeds, seeds that are near each other are collapsed into single seed points at their median, and are treated as single points called “compressed seeds.” This dramatically reduces computing times. From each “compressed seed,” the algorithm expands outwards up to a maximum (user-defined) DMR size, then identifies the smallest expansion containing over 90% of the most variable sites. Following expansion, the tails of these DMRs are trimmed to remove stretches of sites with eigenvector scores similar to those of the background. Final DMR significance is calculated by comparing the rank of all DMR sites to those in a bootstrapped, randomly selected local background.

This algorithm processes relatively quickly (Fig. 1B). When compared to other common DMR calling algorithms in simulated datasets, it shows the greatest accuracy: both in terms of total DMRs called, and at the level of individual bases within DMRs (Fig. 1C, Supplementary Fig. 3B and C). DMR calling in biological datasets also performs as expected (Supplementary Fig. S3D).

Notably, we also tried to benchmark the software Metiline (Jühling *et al.* 2016). However, our testing paradigm involves using the default settings of all softwares, but our simulated genomes can only be processed by Metiline if its settings are altered. Once modified, Metiline does report similar results to PCBS in our simulated genomes, showing relative resistance to false positives, and a slight inclination to false negatives. It is also quite fast, though because its speed does not scale with genome size as in all the other tested softwares, we cannot representatively benchmark its speed. In the true mouse data, PCBS and Metiline both produce distinct sets of equally robust DMRs, suggesting that in true genomes both may be more prone to false negatives than suggested by simulated datasets.

### 3.3 DMR seeds

Simulations demonstrate that this algorithm is highly resistant to false positives regardless of input seed number (Fig. 1D). However, to reduce the number of false negatives, some consideration must be given when defining the number of seeds for DMR calling. If too few seeds are queried, DMRs around more weakly significant loci will be missed. Conversely, if too many seeds are included, seeds can become “overcompressed.” Overcompression occurs when seeds from multiple nearby DMRs are compressed into a single point, causing the algorithm to look for only one DMR in a region where multiple are found. In addition, increasing the seed number in mouse data appears to increase processing time exponentially while increasing the DMR calls logarithmically (Supplementary Fig. S3E). Thus, for any dataset, the optimal seed number strikes a balance between including too few seeds and data overcompression. While we do offer some functionality to help safeguard against overcompression, we generally recommend using a seed number 1%–3% of the total filtered loci number to optimize the true-positive call rate against computing times.

### 3.4 Additional functionality

In addition to PCBS’s DML and DMR calling functionality, the package offers tools for broader analyses. PCBS contains in-built functions that enable users to directly query regions of interest for differential methylation by comparing the eigenvector scores of loci within a region to those in the local background, taking all methylated loci in the genome into consideration without noticeable effects on computing time. This allows users to easily and directly assess the exact methylation levels at regions of interest in a simpler and more foolproof manner than looking for overlaps with DMRs. PCBS also offers functionality to create metagene plots of input regions. Like other PCBS functions, these can be generated remarkably quickly without the need to remove low-variability loci.

## 4 Conclusion

PCBS has two notable limitations. The first being that it only offers single-factor comparisons between two conditions. However, nothing about PCBS’s underlying logic precludes future development of more complex comparisons, and the speed and accuracy of PCBS in its current form is very promising.

The second limitation of PCBS is that it does not generate significance values at the level of individual loci. However, because analysis with PCBS aims to examine the genome holistically, identifying differentially methylated loci is less important. While a simple rank cut-off performs comparably to significance-based DML callers, we primarily include this comparison as a proof of concept to show that the eigenvector scores upon which we base our DMR calling are accurate at identifying actual differential sites. Still, for users who wish to focus exclusively on DMLs for their analysis, we suggest using DSS if computing resources are a concern, or DNMTools’ radmeth function if they are not, as these softwares will give marginally more accurate results in addition to locus-level significance values.

In addition, some users may be concerned about the potential for false negatives with PCBS. In this case, we suggest combining DMRs called by PCBS and Metiline, and running downstream analyses using PCBS’s user-friendly framework.

Despite these limitations, PCBS demonstrates short computational times, sensible DMR calling in archived mouse WGBS data, and high-fidelity DMR calling in simulated datasets. PCBS additionally introduces novel functionality that improves the flexibility of WGBS analyses. Altogether, PCBS is a powerful new option for bisulfite sequencing analysis.

## Supplementary Material

btae593_Supplementary_Data

## Data Availability

The archived data underlying this article are available in GEO under the accession number GSE89275; this dataset was derived from sources in the public domain: doi:10.1186/s13059-017-1185-3.
